# COVID-19, Fiscal Federalism and Provincial Debt: Have We Reached a Critical Juncture?

**DOI:** 10.1017/S0008423920000621

**Published:** 2020-06-19

**Authors:** Kyle Hanniman

**Affiliations:** Department of Political Studies, Queen's University, Mackintosh-Corry Hall, Room C321, 68 University Ave, Kingston, Ontario, K7L 3N6

## Abstract

In 2019, Canada's gross subnational debt to GDP was well over 40 per cent, easily the highest in the world (see Figure 1). This level will only grow as the provinces grapple with the pandemic and its fiscal effects. Some believe surging provincial debts have brought Canadian federalism to a critical juncture: they have greatly increased the odds of federal measures to stabilize provincial finances. This article assesses this claim. The cleanest and most balanced path to fiscal sustainability is a combination of enhanced federal transfers, which would bolster provincial fiscal capacity, and national fiscal rules, which would constrain provincial borrowing. But the former is unlikely to restore sustainability on its own, and the latter would require a severe provincial debt crisis, which Canada's existing fiscal federal structures can avoid. COVID-19 has increased the odds of certain reforms, and it is difficult to predict their long-run effects. But any obvious paths to fiscal sustainability remain hidden.

## Introduction

**Figure 1:**
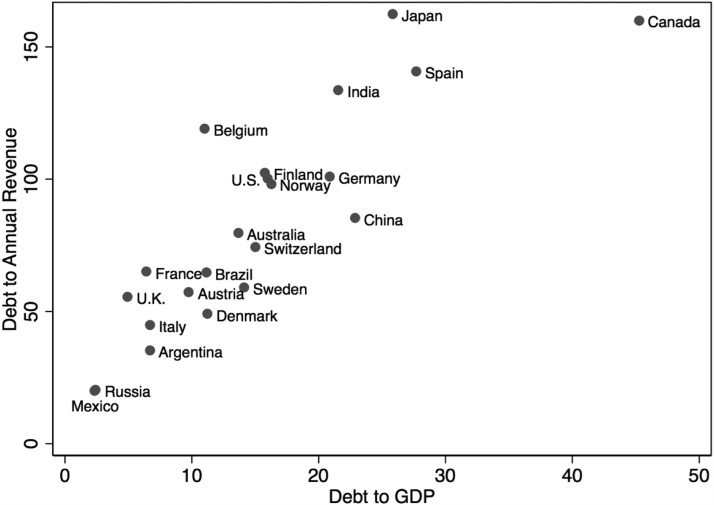
Gross subnational debt to revenue and GDP, 2019 (percentage). Source: S&P. Debt refers to entire subnational sector.

In 2019, Canada's gross subnational debt to GDP was well over 40 per cent, easily the highest in the world (see Figure 1).^1^ This level will only grow as the provinces grapple with the pandemic and its fiscal effects. Some believe surging provincial debts have brought Canadian federalism to a critical juncture: they have greatly increased the odds of federal measures to stabilize provincial finances. This article assesses this claim. The cleanest and most balanced path to fiscal sustainability is a combination of enhanced federal transfers, which would bolster provincial fiscal capacity, and national fiscal rules, which would constrain provincial borrowing. But the former is unlikely to restore sustainability on its own, and the latter would require a severe provincial debt crisis, which Canada's existing fiscal federal structures can avoid. COVID-19 has increased the odds of certain reforms, and it is difficult to predict their long-run effects. But any obvious paths to fiscal sustainability remain hidden.

## Why Do Provinces Borrow So Much?

The sources of the provinces’ exceptional indebtedness are many, but three stand out: (1) their large, rigid and open-ended expenditures (especially healthcare); (2) their cyclical revenue streams (including income tax, sales tax and resource royalties); and (3) their ability to borrow without federal restrictions at low interest rates. Condition 1 puts spending under steady upward pressure. Conditions 1 and 2 make provincial budget balances vulnerable to shocks. And Condition 3 allows provinces to finance structural and cyclical shortfalls with debt. (Some would add inadequate federal transfers to this list, but increasing transfers is not a necessary condition for stabilizing provincial debt, though it may be a necessary condition for stabilizing it in a desirable way. US states also struggle under conditions 1 and 2, but cannot borrow to the same degree, because of widespread balanced-budget legislation and vigilant credit markets. Thus, a harder budget constraint could also lower provincial debts, but at considerable economic and social cost, particularly during recessions.)

But how, if provincial debts are so high, do provinces manage to borrow so cheaply? One reason is the secular plunge in global interest rates. Another is the assumption, widely held among investors, that Ottawa is unlikely to let a province default (Hanniman, 2018). This assumption does not elevate provinces to the status of a federal borrower, but it does increase their borrowing capacity significantly.

## Can the Provinces’ High Debt Levels Persist?

**Figure 2:**
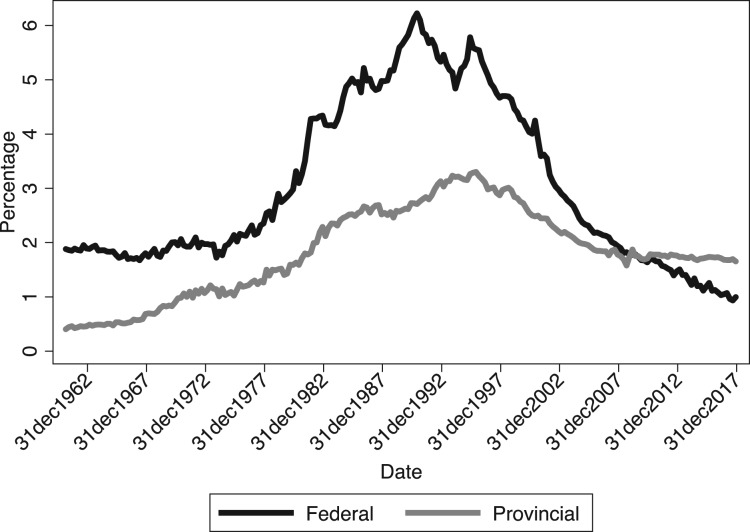
Interest payments as a percentage of GDP. Source: S&P.

Provincial debts cannot grow forever. Eventually, provinces will have to adjust. But as Figure 2 suggests, current pressure is not as powerful as many assume. Provincial debts are higher than they have ever been, but interest payments to GDP are well below their historical peak. Still, the trajectory of provincial debt is unnerving. Growing debt implies future tax increases and expenditure cuts, as interest payments slowly eat into program expenditures. The risk of market-induced austerity is also higher provincially, where economic growth rates are less likely to exceed interest rates and credit shocks are more frequent.

But why, if bailout expectations are high, do provinces not borrow on federal terms? The answer lies in part in the provinces’ place in the global financial cycle. Investors rebalance their portfolios toward less risky and more liquid assets during periods of global financial distress. Subnational bonds are riskier than sovereign debt. (There is always some possibility, however low, that the federal government will not come to a teetering province's rescue.) They are also less liquid. Accordingly, their relative value declines when market conditions deteriorate. These “flights to quality” and “liquidity” cause intergovernmental spreads to diverge. They can also make it difficult for provinces to borrow. With the market's desired spread rising and secondary trading in provincial bonds thinning, provinces and their underwriters (who buy the debt and resell it to investors) struggle to find a market-clearing price (a problem far bigger for provinces with small and illiquid pools of debt than it is for Ontario).

In theory, markets are never closed. Provinces can borrow as long as they pay a premium over unreliable spread indications from secondary markets. Perhaps but provinces have been known to sit out these periods and issue short-term debt instead. This increases refinancing risk, but keeps borrowers from spooking investors with desperate spread concessions. These time-outs are usually short, but not always. Newfoundland and Labrador (NL) went six months without issuing a bond in 2015–2016, despite significant borrowing need (Hanniman, 2018).

If, prior to the pandemic, there was an immediate threat to provincial borrowing, it was this: markets were generally happy to lend to provinces but would occasionally recoil or demand higher spreads in the face of global liquidity shocks. These shocks have been too short to trigger a repayments crisis. But perhaps all that was needed was a national recession to amplify their effects—something that would send deficits and provinces’ liquidity and credit premiums soaring.

## The COVID-19 Shock

From late February to early March, the economic and financial gravity of the pandemic began to become evident. Stock markets plunged and the provinces' bond spreads spiked. Resource-based provinces (also reeling from plunging oil prices) were hardest hit. NL was briefly unable to borrow. British Columbia (with its triple-A rating) and Ontario and Quebec (with their liquid pools of debt) fared best. But all provinces saw their 10-year bond spreads increase 66 to 102 basis points in a month—a faster increase than anything observed during the 2008 crisis.

And yet the provinces have weathered the crisis reasonably well. All-in borrowing costs have fallen^2^; all provinces, including NL, have managed to issue long-term debt; and borrowing has been proceeding at a rapid pace. Demand for provincial bonds has been shaky at times, but the Bank of Canada has committed to buying up to 40 per cent of each of the provinces’ short-term debt offerings through the Provincial Money Market Purchase (PMPP) program and $50 billion of bonds with a maturity of 10 years or less through the Provincial Bond Purchase Program (PBPP).^3^ These interventions have helped stabilize the market, but they are liquidity, not solvency, measures. They do not address the provinces’ long-term challenges.

## Transformative Solutions?

Many believe surging provincial debts have greatly increased the odds of meaningful federal efforts to stabilize them. The pandemic may, in other words, have brought Canadian federalism to a critical juncture.^4^ Definitions of critical junctures vary, but we often think of them as moments in which the structural constraints on political action are briefly but substantially relaxed, "with two main consequences: the range of plausible choices open to powerful political actors expands substantially and the consequences of their decisions for the outcome of interest are potentially much more momentous” (Keleman and Capoccia, 2007: 343). Have we really reached this point with respect to provincial debt?

I approach this question with great humility. The federal government is still coping with the pandemic and may not turn to the provinces' long-run challenges for some time. We have yet, in other words, to reach a critical choice point, and a lot could happen before we do. We also want to avoid the pitfall of radically dividing history into periods of institutional stability and change. Like all evolutionary systems, Canadian federalism is in a constant state of adaptation, and it is often the accumulation of small choices (to say nothing of their unexpected effects) that have the biggest long-term impact. Still, the literature has identified a number of paths the federal government could take to restore provinces’ fiscal sustainability, and I will consider the political and technical potential of two. The first, reforming the transfer system, is popular in Canada's academic and policy circles. The second, national fiscal rules, is widely discussed in the comparative federalism literature.

### Reforming the Transfer System

Many believe that increasing federal transfers could significantly lower provincial debts. Proposals fall under two categories: temporary measures to address the pandemic and lasting measures to address enduring challenges. The latter include a larger and needs-based Canadian Health Transfer to offset rising healthcare costs and an enhanced Fiscal Stabilization Program (FSP) to smooth provincial revenue shocks (Béland et al., 2020). These proposals could go a long way toward realigning fiscal capacities and responsibilities. But how likely is the federal government to implement them?

The federal government has already announced $14 billion to help provinces and territories reopen their economies. This will provide significant relief, but it will not be nearly enough to stabilize provincial debt. The provinces' fiscal woes preceded the crisis, and the announcement falls well short of their net borrowing requirements. Thus, the provinces may need additional short-term support and long-term transfer reform.

There is good reason to think the latter is coming. Ottawa was poised to enhance the FSP before the crisis, and the current situation will only strengthen calls to offset provincial revenue shocks. The pandemic has also piqued federal interest in long-term care and other areas of provincial jurisdiction, and Ottawa's aggressive fiscal response in recent months may have raised popular expectations of its social policy role. But there is also reason to believe the response will be restrained. Several provinces have already voiced their opposition to federal conditions, and that may affect the support Ottawa is willing to provide. Ottawa may also come under pressure to balance its own budget, if not from bond markets (interest rates remain low), then from political forces. Material measures to bolster provincial fiscal resources are not a given.

Nor are they guaranteed to balance provincial budgets. Comparative evidence shows increasing transfers often increases deficits, particularly if supports shield (or are perceived to shield) borrowers from irresponsible choices (Rodden, 2006). Pandemic-related transfers are temporary and unlikely to create this perception. And Ottawa can mitigate moral hazard, as it generally does, by allocating transfers according to clear and exogenous criteria that opportunistic provinces cannot renegotiate or game. But Ottawa will also face pressure to provide ad hoc supports (such as the Atlantic Accords), and no amount of fiscal engineering is likely to significantly alter the market's bailout expectations. Bond markets will continue, therefore, to let provinces borrow more than they can sustain. Additional transfers may increase the capacity to balance budgets, but without appropriate incentives, they may not be enough.

### National Fiscal Constraints

In his landmark book, Jonathan Rodden describes two mechanisms for managing subnational debts: a market-based approach, in which bondholders contain deficits with higher risk premiums, and a hierarchical approach, in which the central government does the disciplining (Rodden, 2006). Often hierarchy is a consequence of failed market surveillance. Investors allow units to borrow more than they can repay, because they expect the center to bail them out. A crisis emerges and the bailout comes, but in an effort to limit moral hazard, the centre demands or negotiates a degree of fiscal restraint. Fiscal rules have yet to emerge in Canada despite the provinces’ periodic market struggles. Why?

For starters, Canada is a deeply federal society with powerful regional identities. A conditional bailout would be met with about as much enthusiasm as a structural adjustment from the International Monetary Fund. The provinces and federal government have generally tried, therefore, to avoid it. In 1936, Alberta's Social Credit government took avoidance to the extreme, opting to default rather than accept the supervision of a federal loans council (a condition of the next bailout).

A second obstacle is institutional. Renegotiating intergovernmental burdens is challenging in any federation. But certain institutions—notably a vertically integrated party system—help facilitate distributional bargains (Wibbels, 2005). Canada lacks this institutional machinery.

None of these obstacles would matter if provinces faced a prolonged repayments crisis. They would have to accept Ottawa's dictates or default. But it is not clear, outside of the Great Depression, when that moment would have been. Saskatchewan flirted with default in 1993, but quickly turned it around, first with a small and unconditional bailout, which allowed it to maintain its investment-grade credit rating (MacKinnon, 2003), and then with austerity, which was motivated by the fear of requiring a larger and thus conditional level of support (Poitras, 2018). This combination (austerity and just enough federal support to see a province through) is an effective way of avoiding the centralizing alternative.

## Conclusion

The pandemic has put enormous pressure on provincial budgets. It has also increased pressure for federal solutions. Changes to basic transfer programs seem increasingly likely, suggesting we have reached a critical juncture in this narrow sense. But the most obvious path to fiscal sustainability (a combination of additional transfers and national fiscal rules) likely remains closed.^5^ Stabilizing provincial debt will likely require creative solutions.

## Notes

1 If we look at gross debt to revenue, arguably a better indicator of fiscal sustainability at the subnational level, Canada's subnational sector is second only to Japan's (see Figure 1).

2 This is because federal rates have fallen further than provincial spreads have risen.

3 The sector was borrowing at a record clip before the PBPP was announced, but under the highly volatile market conditions provinces usually try to avoid (Hanniman 2020).

4 Béland et al. (2020) also examine whether COVID-19 has brought fiscal federalism in Canada to a critical juncture, but their discussion of outcomes and fiscal arrangements is broader than my focus on provincial debt.

5 Though NL's fiscal situation may be the first to test that assumption.
